# Audiometric and Vestibular Function after Classic and Reverse Stapedotomy

**DOI:** 10.3390/medicina60050803

**Published:** 2024-05-13

**Authors:** Janez Rebol, Petra Povalej Bržan

**Affiliations:** 1 Department of Otorhinolaryngology, University Medical Centre Maribor, Ljubljanska Ulica 5, 2000 Maribor, Slovenia; 2 Faculty of Medicine, University of Maribor, Taborska Ulica 8, 2000 Maribor, Slovenia; petra.povalej@um.si; 3 Faculty of Electrical Engineering and Computer Science, University of Maribor, Koroska Cesta 46, 2000 Maribor, Slovenia

**Keywords:** stapedotomy, reverse stapedotomy, otosclerosis, argon laser

## Abstract

*Background and Objectives:* Besides classical stapedotomy, reverse stapedotomy has been used for many years in the management of otosclerosis. Our study aims to investigate whether reversing the surgical steps in stapedotomy impacts vestibular function and hearing improvement. *Materials and Methods:* A cohort of 123 patients underwent either classic or reverse stapedotomy procedures utilizing a fiber–optic argon laser. Audiological assessments, following the guidelines of the Committee on Hearing and Equilibrium, were conducted, including pure tone average, air–bone (AB) gap, overclosure, and AB gap closure. Vestibular evaluation involved pre- and postoperative comparison of rotatory test parameters, including frequency, amplitude, and slow phase velocity of nystagmus. *Results:* The study demonstrated an overall median overclosure of 3.3 (3.3, 5.0) dB and a mean AB gap closure of 20.3 ± 8.8 dB. Postoperative median AB gap was 7.5 (7.5, 11.3) dB in the reverse stapedotomy group and 10.0 (10.0, 12.5) dB in the classic stapedotomy group. While overclosure and AB gap closure were marginally superior in the reverse stapedotomy group, these differences did not reach statistical significance. No significant disparities were observed in the frequency, slow phase velocity, or amplitude of nystagmus in the rotational test. *Conclusions:* Although not always possible, reverse stapedotomy proved to be a safe surgical technique regarding postoperative outcomes. Its adoption may mitigate risks associated with floating footplate, sensorineural hearing loss, and incus luxation/subluxation, while facilitating the learning curve for less experienced ear surgeons.

## 1. Introduction

Otosclerosis, characterized by localized otic capsule pathology involving alternating phases of bone resorption and formation, stands as a predominant cause of hearing loss in Europe and the USA. Histopathologically, the otosclerotic process is characterized by abnormal bone remodeling, involving the replacement of the otic capsule bone with a hypercellular woven bone, which may undergo further remodeling to finally reach a mosaic sclerotic appearance. The site of predilection is the fissula ante fenestram, which lies anterior to the stapes footplate. Otosclerotic foci may also appear in other sites, even in the region of the round window and cochlea. If the otosclerotic process begins to develop anterior to the footplate, the footplate becomes displaced posteriorly, resulting in low-frequency conductive hearing loss. If the whole footplate is fixated, conductive hearing loss takes place in all frequencies [1]. While relatively rare in developing countries and the Japanese population, its impact on patients manifests through progressive hearing deterioration, often hearing better with background noise [2]. This paradox is known as paracusis of Willis. Classical audiometric manifestations include low-frequency conductive hearing loss, Carhart notch, type A or As tympanogram, biphasic or absent stapedius reflex, and negative Rinne test. Despite extensive research, the etiology of otosclerosis remains elusive. Surgical intervention, when successful, holds considerable satisfaction for both patients and surgeons.

**Figure 1:**
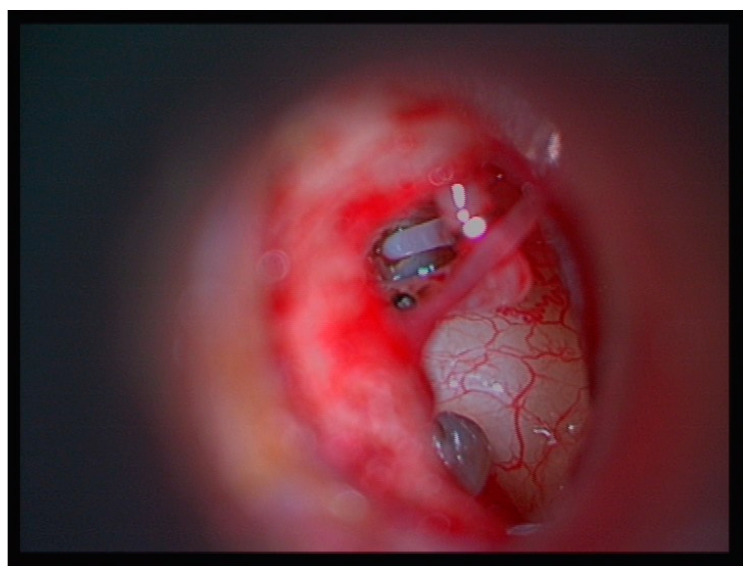
Right ear. The tympanomeatal flap is raised. The Fish prosthesis is crimped onto the long process of the incus. The superstructure of the stapes is still intact.

**Figure 2:**
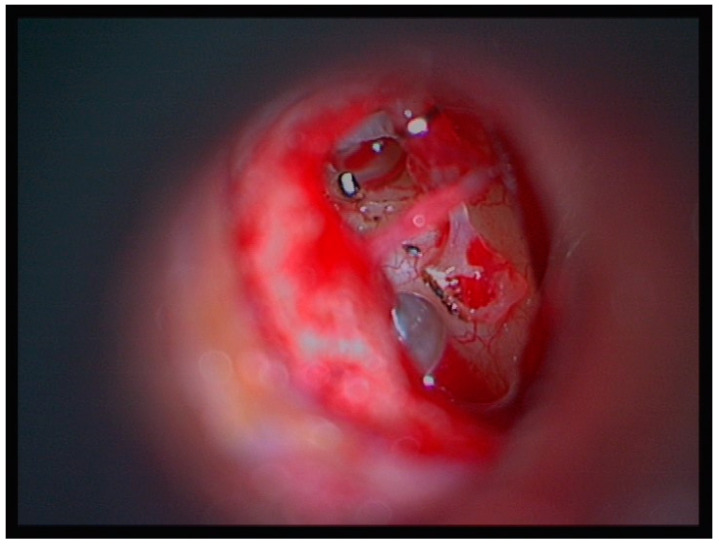
The stapes superstructure is removed from the footplate and placed on the promontory. The Fisch prosthesis is still in place.

The advent of stapedectomy in 1956 by Shea marked a significant milestone in otosclerosis management, evolving into stapedotomy techniques over time [3]. Notably, Fisch introduced modifications in stapedotomy steps in 1980. He proposed stapedotomy of the footplate while keeping the stapes suprastructure (SSS) and incudostapedial joint (ISJ) intact, followed by the disjunction of the ISJ and removal of the SSS prior to prosthesis insertion to mitigate the risk of floating footplate or incus dislocation [4]. Fisch suggested first creating the stapedotomy hole in the footplate, followed by inserting a 0.4 mm Teflon piston into the footplate and compressing it to the long process of the incus. The stapes structure is then removed (Figure 1 and Figure 2), prioritizing a smaller hole and reduced inner ear exposure to potentially minimize postoperative complications. Further refinements, such as posterior crus removal to enhance surgical visibility and facilitate fenestration, have since been introduced [5].

In light of these advancements, our study aims to evaluate whether reversing surgical steps in stapedotomy enhances complication rates and hearing outcomes. Through a comparison of audiological and vestibular results following stapedotomy, we aim to elucidate the efficacy and potential benefits of reverse stapedotomy.

## 2. Materials and Methods

### 2.1. Sample

A consecutive sample of otosclerotic patients who underwent otosclerosis surgery between June 2018 and May 2020 was included in the study. The study protocol was approved by the local ethical committee, and all participants provided informed consent in accordance with the Declaration of Helsinki. Participants underwent preoperative and postoperative audiometric and vestibular assessments.

### 2.2. Audiometric Assessment

Audiometric evaluations adhered to the guidelines of the Committee on Hearing and Equilibrium [6]. Measurements were conducted one day preoperatively and approximately six weeks postoperatively to assess for overclosure or presumed surgical damage. Pure tone averages were calculated using frequencies of 0.5, 1, 2, and 3 kHz. The air–bone gap (ABG) at these frequencies was determined as the difference between the four-tone pure tone average for air conduction (AC) and the same average for bone conduction (BC). Overclosure or operative damage to hearing was assessed by comparing preoperative and postoperative high pure tone BC averages at 1, 2, and 4 kHz. ABG closure was determined as the difference between preoperative and postoperative ABG.

### 2.3. Vestibular Assessment

The rotational test was employed to assess vertigo, utilizing sinusoidal harmonic acceleration with rotational stimuli ranging from 0.01 to 1.28 Hz to the right and left. The speed of the chair was set to 60 degrees/s. The rotational stimulus at a given frequency was repeated for several cycles. Slow phase velocity, frequency, and amplitude of nystagmus were observed before stapedotomy and on the day after surgery. Statistical analyses involving the rotational test included data from 119 patients.

### 2.4. Surgical Technique

Surgery was conducted under general anesthesia via a transcanal approach, assisted by argon laser. In reverse stapedotomy, the chorda tympani was mobilized following tympanomeatal flap elevation. The footplate was opened with a drill with a diameter of 0.5 mm, and a 0.4 mm-diameter Fisch prosthesis (Teflon platinum) was inserted and crimped onto the long process of the incus. Before the drilling of the stapedotomy, a rosette was created with the laser, which enabled easier drilling of the stapedotomy. The stapedial muscle and posterior crus of the stapes were coagulated with the laser. The stapedial muscle could also be cut with scissors, but we used the laser to avoid damaging the chorda tympani nerve, which is sometimes located above the stapedial muscle. The incudostapedial joint was separated, and the anterior stapedial crus was fractured before removing the stapes suprastructure. Classic stapedotomy was performed in cases where stapedotomy with the drill was not feasible due to a narrow oval window niche with the stapes suprastructure present.

### 2.5. Statistical Analysis

Statistical analysis was conducted using SPSS V29. Numerical variables were described with mean ± standard deviation or median (95% confidence interval) for non-normal distributions. The Kolmogorov–Smirnov test or Shapiro–Wilk test assessed data distribution. Categorical variables were described with frequencies and percentage values. Paired samples *t*-tests or Wilcoxon signed rank tests compared pre- and post-surgery measurements, while differences between reverse and classic stapedotomy were analyzed using Student *t*-tests or Mann–Whitney U tests. The Levene test evaluated equality of variances, and Pearson’s Chi-Square test compared the frequency of positive differences in rotational tests between stapedotomy groups. Statistical significance was set at *p* < 0.05.

## 3. Results

A total of 123 patients were included in the study, comprising 56.9% females and 43.1% males, with a mean age of 47.4 ± 10.2 years. The distribution of surgery side was nearly equal, with 63 patients (51.2%) undergoing surgery on the left side and 60 patients (48.8%) on the right side. Reverse stapedotomy was performed in 51 patients (41.5%), while classical stapedotomy was employed in 72 patients (58.5%). There were no significant differences in demographic characteristics between the two groups of patients (Table 1).

### 3.1. Hearing Results

**Figure 3:**
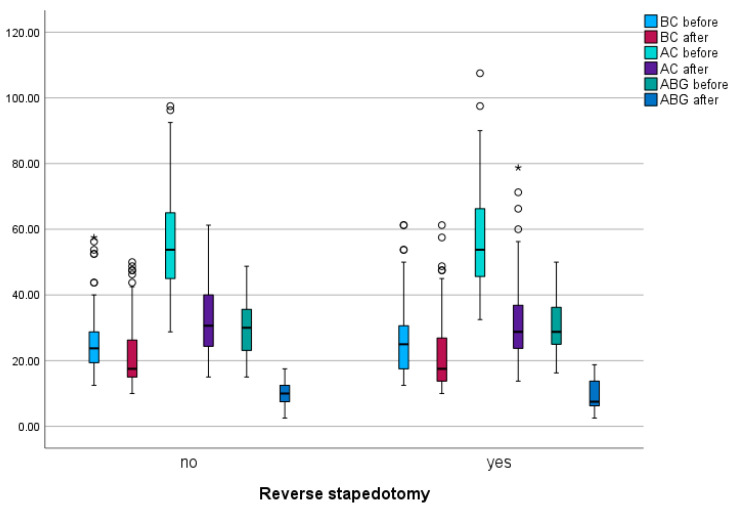
Pre- and postoperative BC, AC, and ABG (classic and reverse stapedotomy). * *p* < 0.001.

The results of bone conduction (BC), air conduction (AC), air–bone gap (ABG), overclosure, and closure of the ABG are summarized in Table 2. A statistically significant difference was observed in both BC (*p* < 0.001) and AC (*p* < 0.001) before and after surgery (Figure 3). BC improved in 97 patients (78.9%), remained unchanged in 11 patients (8.9%), and slightly deteriorated in 15 patients (12.2%). AC showed improvement in 120 patients (97.6%) and slight deterioration in 3 patients (2.4%).

However, when comparing the reverse and classic stapedotomy groups, no statistically significant differences were found in the changes in BC and AC before and after surgery (*p* = 0.490 vs. *p* = 0.683).

A statistically significant improvement was observed in ABG before and after surgery (*p* < 0.001). All patients exhibited improvement, with a range of 1.3 to 41.3. The mean improvement was 20.3 ± 8.7. There was no significant difference in postoperative ABG improvement between the reverse stapedotomy or classical stapedotomy groups (*p* = 0.256) (Figure 3).

While the results of overclosure and closure of the ABG were slightly better in the reverse stapedotomy group, these differences were not statistically significant.

### 3.2. Rotational Testing

Rotation testing was performed in 120 of 123 patients. Statistical analysis before and after surgery using the paired *t*-test showed no statistically significant difference in the velocity of the slow phase of nystagmus (t = −0.099; *p* = 0.921). Based on the Wilcoxon signed rank test, we concluded that there was no statistically significant difference in the amplitude of nystagmus before and after surgery (Z = −0.754; *p* = 0.451) and that there was no difference in the frequency of nystagmus before and after surgery (Z = −0.928; *p* = 0.353).

We analyzed the difference between slow phase velocity, frequency, and amplitude of nystagmus in patients who underwent reverse or classic stapedotomy. 

In patients who had reverse stapedotomy, there was a positive difference in slow phase velocity in 54.2% and in those who did not have the reverse technique in 44.4%. In those who had a reverse stapedotomy, there was a positive difference in amplitude in 57.1%, and in those who did not have a reverse stapedotomy, it was in 50.7%. In patients who had an inversion, there was a positive difference in frequency in 59.2%, and in those who did not have an inversion, it was in 52.1%. The difference was not statistically significant (Table 3). We observed that there was no difference in vestibular symptoms before and after surgery (*p* = 0.921, *p* = 0.451, *p* = 0.353). Comparing rotation tests, there was no difference in slow phase velocity, frequency, or amplitude of nystagmus after reversible and classic stapedotomy. Age of the patients also had no effect on rotatory testing results.

Rotation testing was conducted in 119 out of 123 patients. The results presented in Table 3 demonstrate no statistically significant difference in the velocity of the slow phase of nystagmus, amplitude, or frequency of nystagmus before and after surgery (*p* = 0.943 vs. *p* = 0.433 vs. *p* = 0.241).

Further analysis compared slow phase velocity, frequency, and amplitude of nystagmus between patients who underwent reverse and classic stapedotomy procedures. No statistically significant differences were observed in rotation tests when comparing these two groups (Table 3).

Among patients who underwent reverse stapedotomy, a positive difference in slow phase velocity before and after surgery was observed in 26 patients (54.2%). In contrast, 31 patients (44.4%) who did not undergo the reverse technique exhibited a positive difference in slow phase velocity. Similarly, a positive difference in amplitude was noted in 28 patients (57.1%) who underwent reverse stapedotomy and in 36 patients (50.7%) who did not. Regarding frequency, a positive difference was observed in 29 patients (59.2%) who underwent reverse stapedotomy compared to 37 patients (52.1%) who did not undergo inversion. However, these differences were not statistically significant (Table 3).

Comparative analysis of rotation tests revealed no differences in slow phase velocity, frequency, or amplitude of nystagmus between patients who underwent reverse and classic stapedotomy procedures.

## 4. Discussion

### 4.1. Advantages and Considerations of Stapedotomy Techniques

In the pursuit of optimizing surgical outcomes and minimizing complications in stapedotomy procedures for otosclerosis, various techniques and prostheses have been developed. The principle of stapedotomy is to form a calibrated hole in the footplate. This can be done with a microdrill, a microhook, and a laser. Before stapedotomy, stapedial crura are separated and the stapes suprastructure removed. The advantages of performing a stapedotomy with a laser (argon, KTP, CO_2_) are the hemostatic properties of the laser “no touch” surgery, which reduces the chance of a floating footplate; the ability to create a precise fenestra without entering the inner ear, thus minimizing the risk of acoustic trauma; and the possibility to fenestrate a floating footplate without the risk of depressing it into the vestibule.

Many studies have been performed to evaluate the audiological improvement after stapedotomy with different prostheses [7,8,9]. The introduction of the reverse stapedotomy technique by Fisch aimed to enhance stability during surgery, thereby mitigating the risk of inner ear damage associated with floating footplates [4]. Fisch used the endaural approach, and we used the transcanal approach through the ear speculum because the skin of the ear canal heals more quickly.

The reverse stapedotomy technique capitalizes on the support provided by the fixed stapes during the creation of the stapedotomy opening, thus reducing the likelihood of footplate displacement into the vestibule. However, challenges such as a narrow oval window niche and an obliterated footplate may compromise the feasibility of this approach. Fisch reported poorer results with this technique using a 0.4 mm piston at 3 weeks, but the results were the same after this delay. The 0.4 mm piston is more suitable for reverse stapedotomy [4,10].

### 4.2. Comparative Outcomes and Techniques

Szymanski used the CO_2_ laser in reverse stapedotomy and found that the use of a laser in combination with reverse stapedotomy is the safest technique. He compared the classical sequence of surgical steps in a group where the hole in the stapes footplate was made before removing the SSS, a group where the stapedotomy was carried out with the manual perforator, and a group where the opening in the footplate was performed with the CO_2_ laser [10]. Regarding the footplate preservation rate (when the stapes footplate was not broken during stapes suprastructure removal), Ueda found that he could preserve the footplate in 72% when the SSS was removed after piston insertion and in 58% when the SSS was removed before piston insertion [11].

In 2008, Fiorino advocated partial reverse stapedotomy, and was able to close the ABG to 10 dB and had no case of sensorineural hearing loss. Early removal of the posterior stapedial crus solves the main obstacle in reverse stapedotomy [5].

Stapedotomy is considered a delicate and challenging procedure for hearing restoration. A learning curve is present, and regular performance of the procedure have been recommended to maintain surgeon proficiency. In order to minimize surgical risks, reverse stapedotomy with a laser should be performed in combination with a self-clipping prosthesis [12]. Still, many laser surgeons do not use the reverse sequence of surgical steps. Hausler used a fiber–optic argon laser with the classical sequence of steps and suggested that creating the perforation in the footplate and inserting the piston before removing the SSS weakens the footplate and predisposes the patient to footplate fractures [13]. With the fiber–optic laser, the structures of the middle ear are more accessible, including the anterior crus of the stapes. Nevertheless, our study, consistent with previous literature, demonstrates comparable outcomes between classic and reverse stapedotomy in terms of air–bone gap closure and bone conduction improvement [5,13]. Postoperative ABG was even less in our reverse stapedotomy group, but the difference in ABG between the groups with and without reverse stapedotomy was not significant.

Regarding vestibular function, few studies have explored post-stapedotomy outcomes, and to our knowledge, none have specifically investigated vestibular assessments following reverse stapedotomy. Our study, albeit limited by its retrospective nature and small sample size, found no significant changes in vestibular parameters post-stapedotomy, suggesting overall stability in vestibular function following surgery.

It is noteworthy that the use of same type of crimping prosthesis throughout our study eliminated bias associated with prosthesis selection, highlighting the safety and efficacy of classic stapedotomy with an argon laser. Surgery was also performed by one surgeon.

Our patients did not report nausea during rotation testing after stapedotomy, and the test was well tolerated. The differences before and after surgery were relatively small and not statistically significant. This may have been due to the gentle argon laser technique.

## 5. Conclusions

In conclusion, while reverse stapedotomy offers theoretical advantages in stabilizing the footplate during surgery, our study suggests that classic stapedotomy remains a safe and effective procedure for otosclerosis management. Still reverse stapedotomy performed with a laser should be advocated whenever possible to minimize the risks of floating footplate, sensorineural hearing loss, and incus luxation/subluxation in the surgery of stapes fixation. By using the reverse stapedotomy with a laser, the learning curve for an unexperienced surgeon may be shortened.

## Figures and Tables

**Table 1:** Demographic data.

		All (*n* = 123)	Reverse Stapedotomy		*p*-Value
			Yes (*n* = 51)	No (*n* = 72)	
Gender	Female	70 (56.9%)	28 (54.9%)	42 (58.3%)	0.705
	Male	53 (43.1%)	23 (45.1%)	30 (41.7%)	
Age		47.4 ± 10.2	47.6 ± 11.5	47.3 ± 9.2	0.864
Affected side	Left	63 (51.2%)	25 (49.0%)	38 (52.8%)	0.681
	Right	60 (48.8%)	26 (51.0%)	34 (47.2%)	

**Table 2:** Hearing testing before and after surgery in classic and reverse stapedotomy.

Measurement		All (*n* = 123)	*p*-Value (Before/After)	Reverse Stapedotomy		*p*-Value (Reverse/Classic)
				Yes (*n* = 51)	No (*n* = 72)	
BC (dB)	Before	23.8 (23.8, 25.0) ^##^	<0.001	25.0 (23.8, 30.0) ^##^	23.8 (23.8, 27.5) ^##^	0.955
	After	17.5 (17.5, 21.3) ^##^		17.5 (16.3, 23.8) ^##^	17.5 (16.3, 21.3) ^##^	0.971
BC difference (dB)	Before–after	3.8 (3.8, 5.0) ^##^		3.8 (3.8, 5.0) ^##^	3.8 (3.8, 7.5) ^##^	0.490
AC (dB)	Before	53.8 (52.5, 56.3) ^##^	<0.001	53.8 (50.0, 56.3) ^##^	53.8 (50.0, 58.8) ^##^	0.910
	After	28.8 (27.5, 32.5) ^##^		28.8 (27.5, 33.8) ^##^	30.6 (27.5, 33.8) ^##^	0.386
AC difference (dB)	Before–after	23.8 (22.5, 26.3) ^##^		23.8 (22.5, 27.5) ^##^	22.5 (21.3, 26.3) ^##^	0.683
ABG (dB)	Before	30.2 ± 8.3 ^#^	<0.001	30.2 ± 8.3 ^#^	30.2 ± 8.4 ^#^	0.986
	After	10.0 (10.0, 12.5) ^##^		7.5 (7.5, 11.3) ^##^	10.0 (10.0, 12.5) ^##^	0.256
Overclosure (dB)		3.3 (3.3, 5.0) ^##^		3.3 (3.3, 5.0) ^##^	3.3 (1.7, 5.0) ^##^	0.869
Closure ABG (dB)		20.3 ± 8.8 ^#^		20.7 ± 7.5 ^#^	20.1 ± 9.6 ^#^	0.702
Closure ABG difference (dB)	before-after	20.3 ± 8.7 ^#^		20.7 ± 7.5 ^#^	20.0 ± 9.5 ^#^	0.673

^#^ Mean ± standard deviation, ^##^ median (95% CI).

**Table 3:** Rotatory testing before and after surgery in classic and reverse stapedotomy.

Nystagmus		All (*n* = 123)	*p*-Value (Before/After)	Reverse Stapedotomy		*p*-Value (Reverse/Classic)
				Yes (*n* = 49)	No (*n* = 70)	
Frequency	Before	−0.8 (−2.8, 1.0) ^##^	0.241	0.0 (−1.9, 4.7) ^##^	−1.1 (−4.3, 1.9) ^##^	0.927
	After	−2.0 ± 12.9 ^#^		−2.6 ± 14.0 ^#^	−1.5 ± 12.2 ^#^	0.669
Frequency difference	Before–after	1.8 ± 17.1 ^#^		1.5 ± 15.9 ^#^	2.1 ± 17.9 ^#^	0.843
Positive frequency difference	Before–after	66 (55.0%)		29 (59.2%)	37 (52.1%)	0.444
Amplitude	Before	−1.3 (−4.8, 2.7) ^##^	0.433	−0.8 (−5.1, 2.8) ^##^	−1.7 (−8.5, 3.2) ^##^	0.791
	After	−4.8 ± 23.7 ^#^		−7.9 (−15.4, −0.6) ^##^	−2.7 (−7.5, 3.6) ^##^	0.272
Amplitude difference	Before–after	2.2 (−4.6, 6.4) ^##^		4.5 (−3.3, 11.6) ^##^	1.6 (−7.0, 5.7) ^##^	0.435
Positive amplitude difference	Before–after	64 (53.3%)		28 (57.1%)	36 (50.7%)	0.487
Velocity of slow phase	Before	−5.3 ± 19.4 ^#^	0.943	−2.8 (−8.5, −0.3) ^##^	−3.2 (−10.0, 1.5) ^##^	0.970
	After	−5.2 ± 24.4 ^#^		−8.8 ± 26.4 ^#^	−2.8 ± 22.8 ^#^	0.188
Velocity of slow phase difference	Before–after	−0.2 ± 25.2 ^#^		3.2 ± 26.6^#^	−2.5 ± 24.1 ^#^	0.231
Positive velocity of slow phase difference	Before–after	57 (48.3%)		26 (54.2%)	31 (44.3%)	0.291

^#^ Mean ± standard deviation, ^##^ median (95% CI).

## Data Availability

Data are contained within the article.
